# Giant coronary artery aneurysms involving more than one coronary artery: case report

**DOI:** 10.1186/s13019-021-01560-5

**Published:** 2021-06-19

**Authors:** Matthew S Khouzam, Nayer Khouzam

**Affiliations:** 1 Loyola University Medical Center, Stritch School of Medicine, Maywood, IL USA; 2 Division of Cardiothoracic Surgery, Advent Health, Orlando, Florida USA

**Keywords:** Coronary artery aneurysm, Aortic aneurysm, Atherosclerosis, Non-ST segment elevation myocardial infarction, Case report

## Abstract

**Background:**

Coronary artery aneurysms are rare findings in patients undergoing coronary angiography. The presence of multiple coronary artery aneurysms located in more than one coronary artery is even more uncommon. The pathophysiology of such aneurysms is unknown, but the majority are often due to atherosclerosis, congenital heart disease, or vasculitis.

**Case presentation:**

We present a rare case of a 78-year-old female patient who presented with unstable angina and non-ST segment elevation myocardial infarction. On coronary angiography, she was found to have three separate 1 cm saccular aneurysms involving the proximal left anterior descending coronary artery. The right coronary artery could not be visualized. Computed chest tomography revealed a 6.6 × 6.3 cm saccular aneurysm of the right coronary artery, and a 4.4 cm fusiform aneurysm of the ascending aorta. The patient gave no history of percutaneous coronary intervention or cardiac surgical procedures. She had a previous history of endovascular stenting of an abdominal aortic aneurysm. The sizable right coronary artery aneurysm showed extrinsic compression of both the right atrium and ventricle with right ventricular hypokinesis. Serological studies for vasculitis were all negative. Pathology of the aneurysm wall revealed calcific atherosclerosis without evidence of vasculitis. The patient underwent subtotal resection of the right coronary aneurysm with ligation of the proximal and distal ends of the right coronary artery and double bypass surgery to the left anterior descending and right posterior descending coronary arteries.

**Conclusion:**

The presence of multiple, large coronary artery aneurysms is very rare. Treatment can be challenging and should be individualized. Surgical treatment is recommended for giant coronary artery aneurysms to prevent potential complications.

## Introduction

Coronary artery aneurysm (CAA) is a rare pathology of the coronary arteries and is present in up to 4.9% of patients undergoing coronary angiography [1]. Although a consensus classification of CAA is lacking, CAA is generally defined as dilations of the coronary artery where the diameter of the distended segment is 150% of the diameter of the adjacent segments [1]. Furthermore, CAA can be termed “giant” if the dilated segment is either > 8 mm in diameter or 400% of the diameter of the adjacent segments [2]. Among those patients with CAA, dilation of the right coronary artery (RCA) is the most common aneurysmal finding followed by dilation of the left anterior descending coronary artery (LAD) [1]. Concomitant aneurysms of both the RCA and the LAD are extraordinarily rare, and when present, are most frequently associated with Kawasaki disease [3]. We present the following case report of a 78-year-old female patient with a history of endovascular stenting of an abdominal aortic aneurysm who presented with unstable angina and non-ST segment myocardial infarction and was found to have three separate giant 1 cm saccular aneurysms involving the proximal LAD as well as a giant 6.6 × 6.3 cm saccular aneurysm of the RCA.

## Case report

**Fig. 1:**
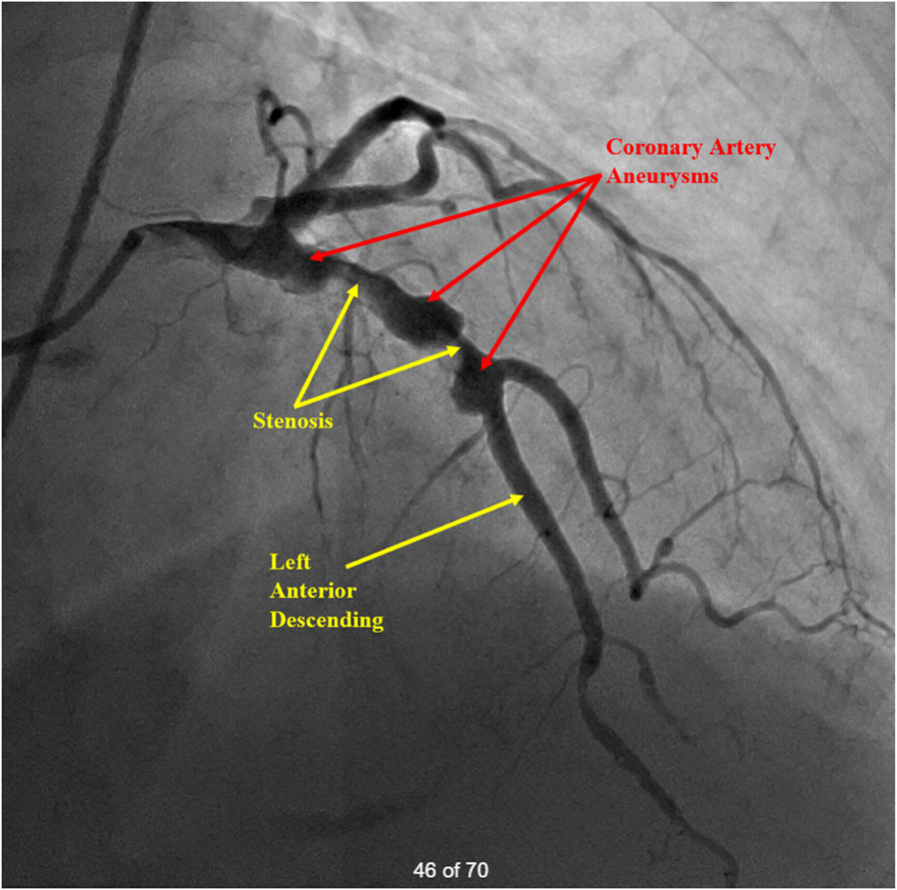
Coronary angiography. Left coronary artery injection demonstrating saccular aneurysms with associated stenoses involving 3 separate regions of the LAD

A 78-year-old Caucasian female with no previous cardiac history presented to an outside hospital with chest and jaw discomfort. Her past medical history is significant for hypertension, atrial fibrillation, endovascular stenting of an abdominal aortic aneurysm, fibromyalgia, and dyslipidemia presented to an outside hospital with chest and jaw discomfort. She was found to have sustained a non-ST segment elevation myocardial infarction. She underwent cardiac catheterization that revealed three separate 1 cm saccular aneurysms of the LAD with associated focal stenosis. The circumflex coronary artery was angiographically free of disease (Fig. 1). The RCA could not be visualized despite multiple attempts. Left ventricular function was preserved. During the procedure, she developed complete heart block necessitating placement of a temporary transvenous pacemaker. However, catecholaminergic support was not needed. In addition, anticoagulative therapy with intravenous heparin was initiated.

**Fig. 2:**
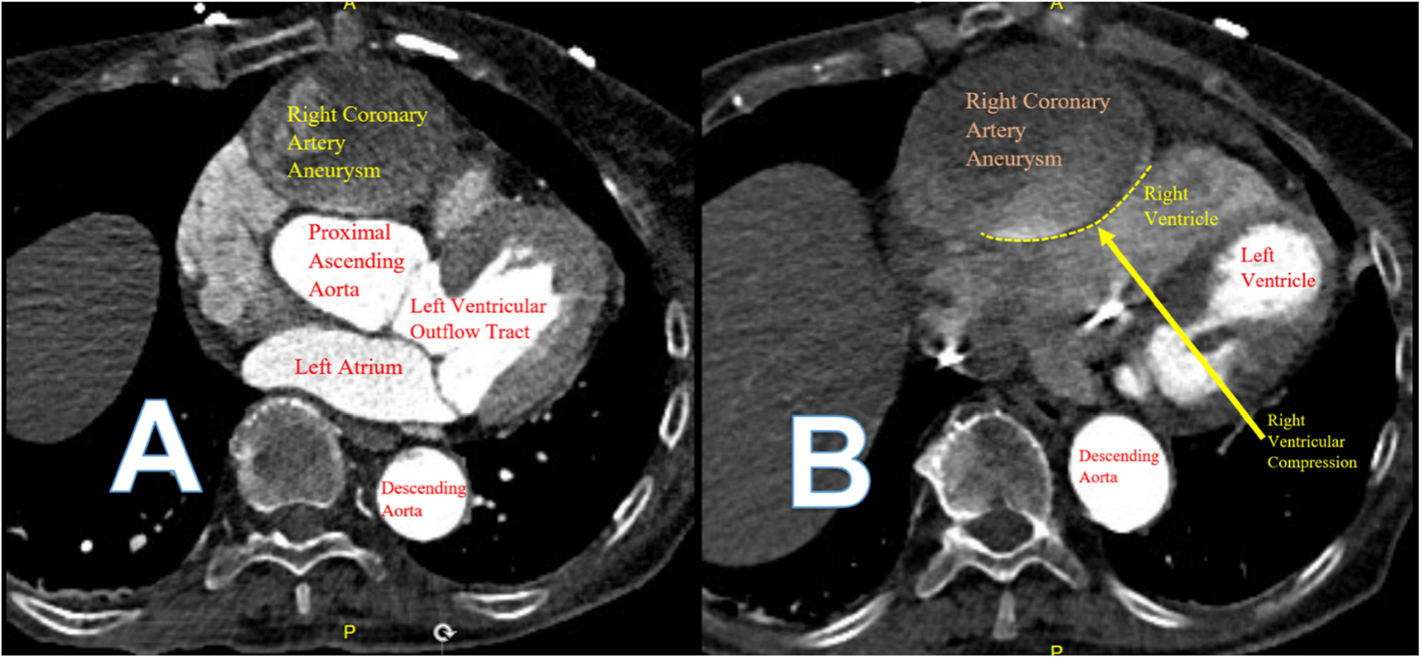
Computed Tomographic Chest Scans. **A** Giant RCAA at the level of the aortic root. **B** Giant RCAA with extrinsic compression of the right ventricle

The patient was transferred to our institution for further evaluation. Upon arrival, she was found to have an elevated troponin of 1.61 ng/ml (normal high less than 0.03 ng/ml). She underwent a computed tomography (CT) coronary angiography with contrast that revealed a 6.6 × 6.3 cm saccular right coronary artery aneurysm (RCAA) extending 8.8 cm in length with turbulent flow seen within the aneurysm. The RCAA compressed both the right atrium and ventricle with evidence of right ventricular hypokinesis (Fig. 2). The distal RCA was poorly visualized but appeared to have some flow. Also noted was a 4.4 cm fusiform ascending aortic aneurysm without leak or dissection. Serological studies (antineutrophil cytoplasmic antibody, immunoglobulin G4, erythrocyte sedimentation rate, C-reactive protein) were all negative.

**Fig. 3:**
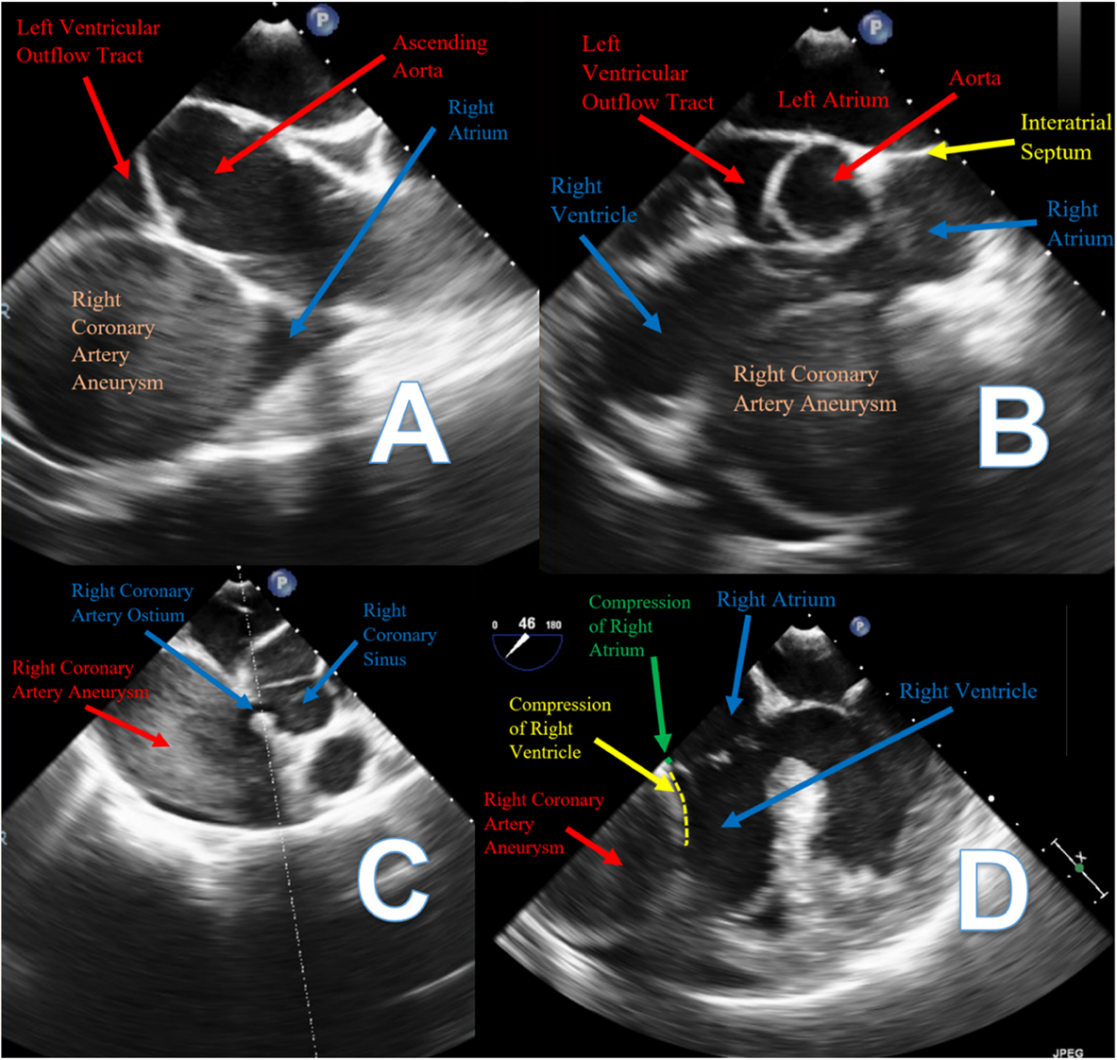
Intraoperative Transesophageal Echocardiograms. **A** Turbulent flow within the giant RCAA. **B** Extrinsic compression of the right ventricle by the giant RCAA. **C** Ostium of the giant RCAA from the right coronary sinus with turbulent flow in the aneurysm. **D** Extrinsic compression of the right atrium and the right ventricle by the giant RCAA

She was taken to the operative suite. Intraoperative transesophageal echocardiogram confirmed the large RCAA arising from the right coronary sinus with turbulent flow in the aneurysm sac with extrinsic compression of the right atrium and ventricle (Fig. 3). There was no evidence of coronary cameral fistulizaton.

**Fig. 4:**
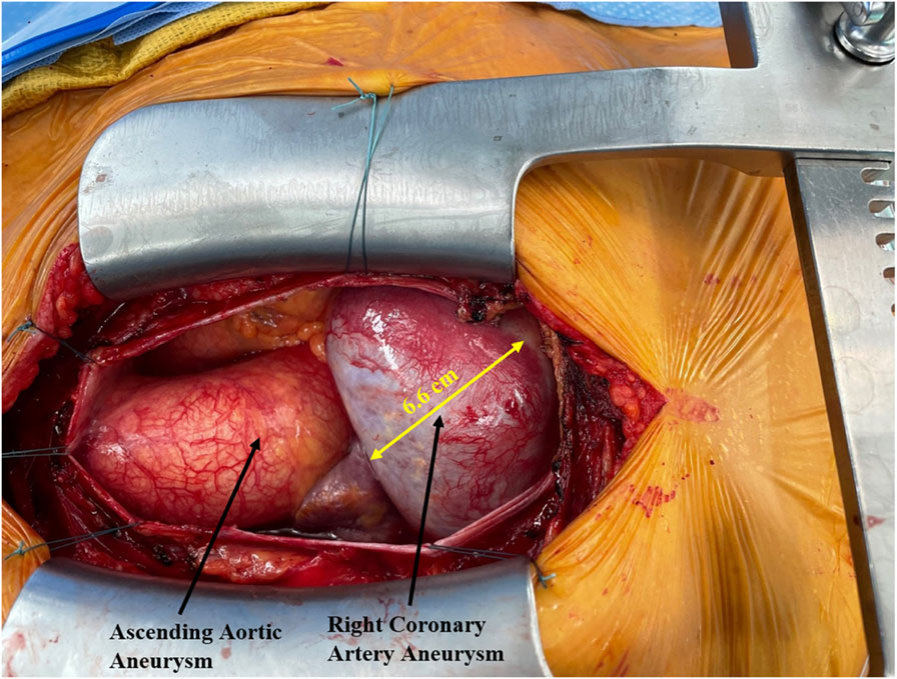
Surgical image. Ascending aortic aneurysm and giant RCAA

**Fig. 5:**
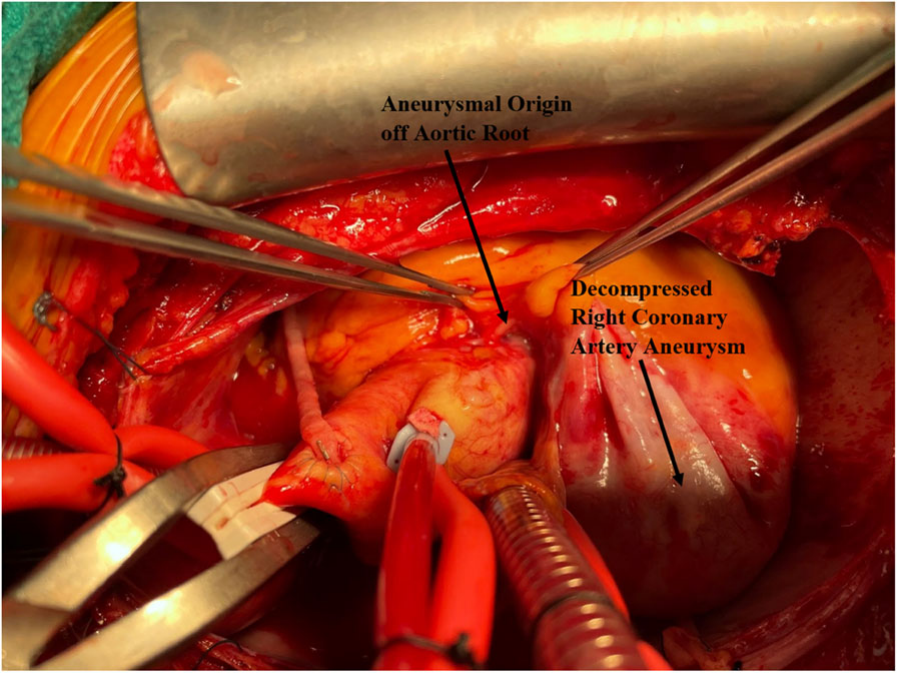
Surgical image. Proximal segment of giant RCAA originating from aortic root

**Fig. 6:**
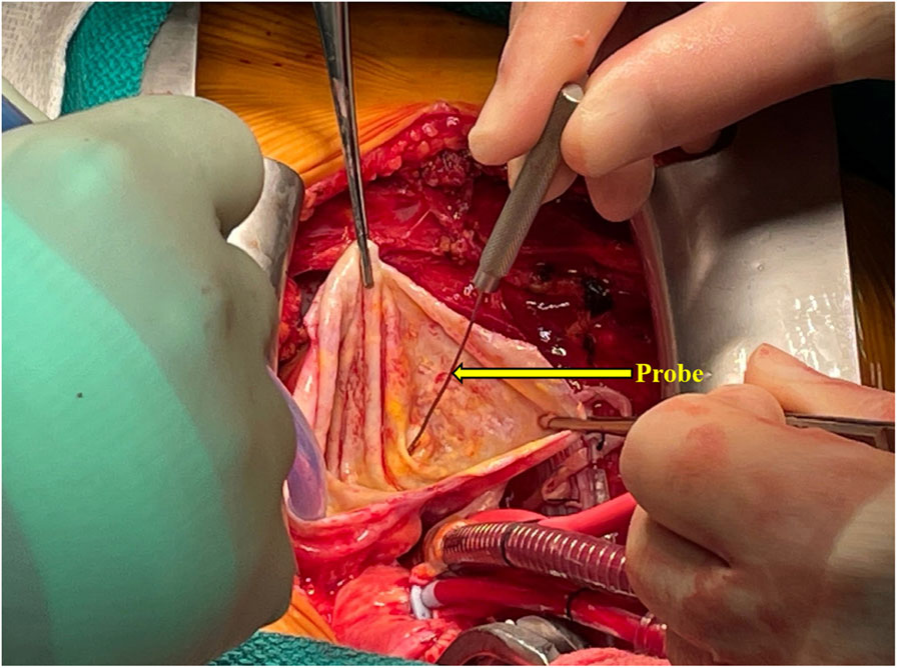
Surgical image. Opened aneurysm cavity with probe in distal RCA

**Fig. 7:**
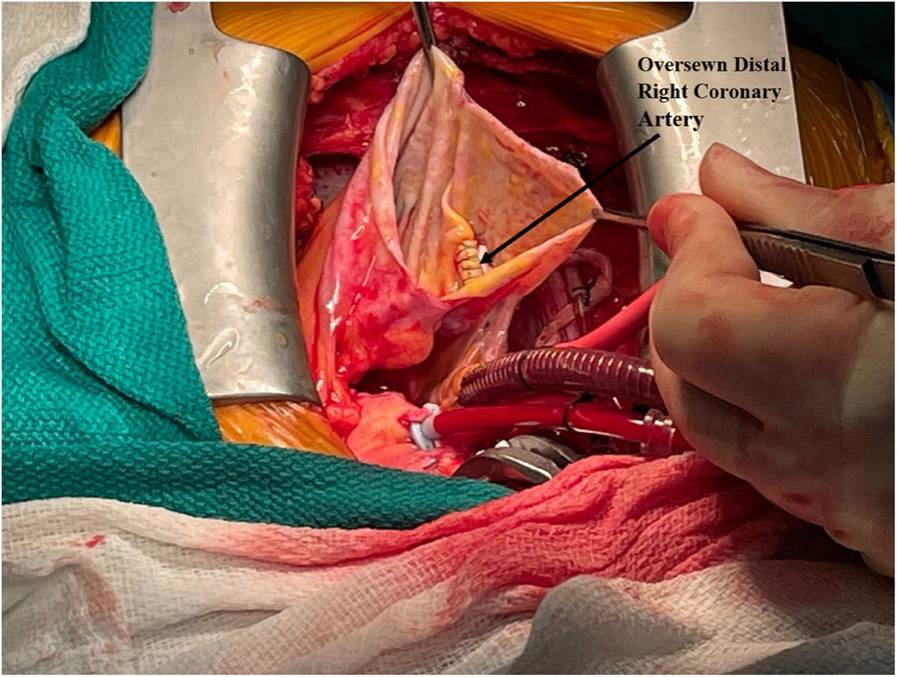
Surgical image. Opened aneurysm cavity with oversewn distal RCA

**Fig. 8:**
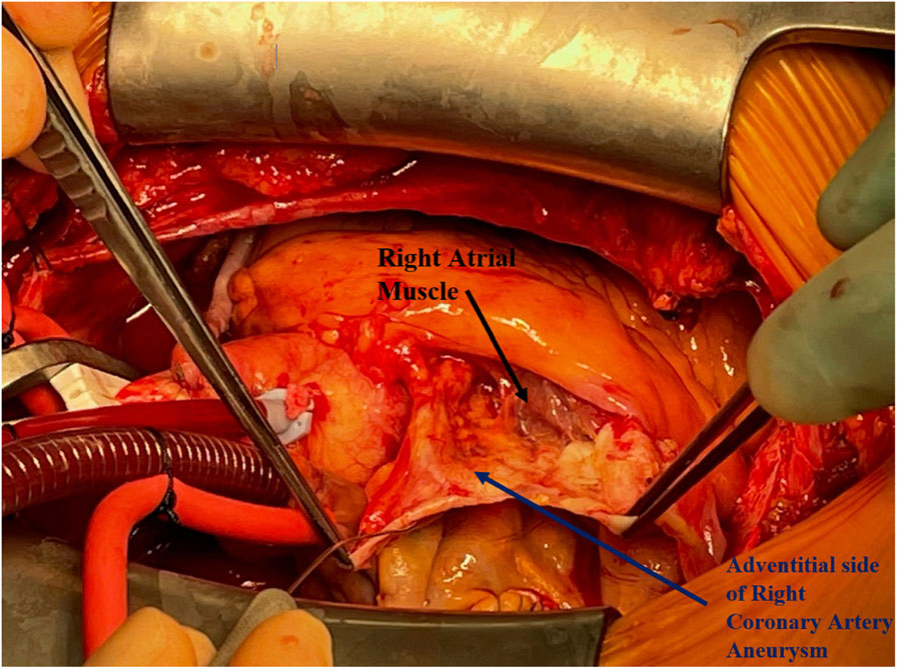
Surgical image. Giant RCAA dissected free from right atrial free wall

**Fig. 9:**
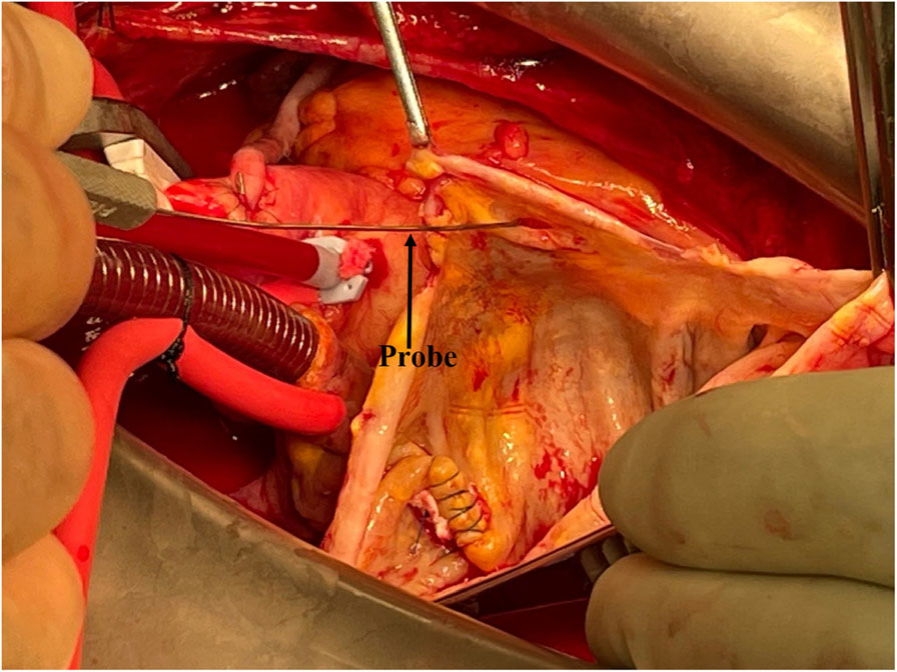
Surgical image. Opened giant RCAA cavity with probe in proximal RCA and oversewn distal RCA

**Fig. 10:**
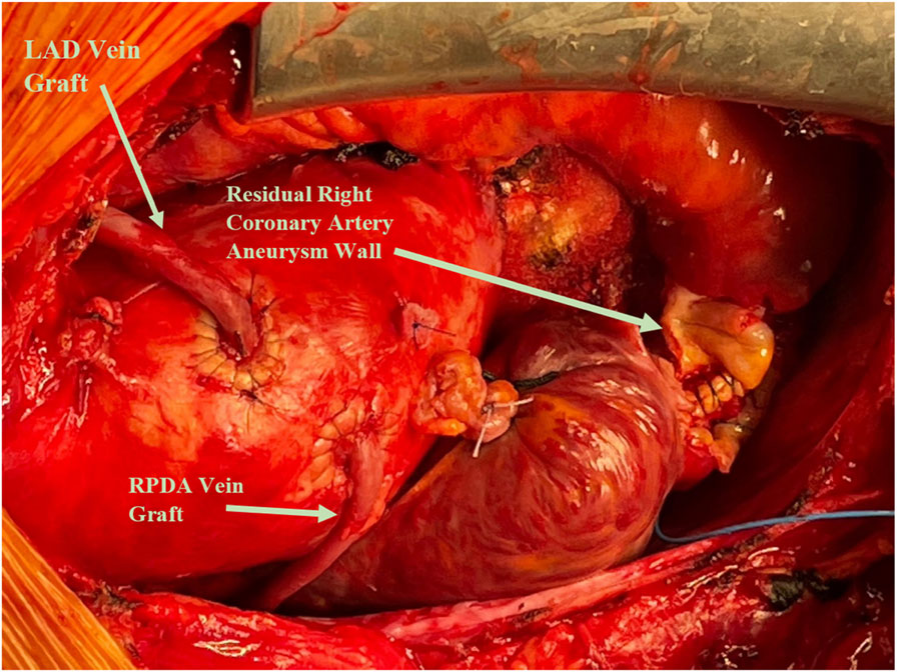
Surgical image. Saphenous vein grafts to LAD and RPDA. Subtotal resection of giant RCAA and oversewn distal RCA

The patient underwent double bypass surgery using saphenous vein grafts to the right posterior descending coronary artery (RPDA) and the LAD. A mammary graft was not used due to concerns regarding potential competitive flow. The right coronary aneurysm was opened and found to have no thrombus. Subtotal resection of the aneurysm wall was performed with oversewing of the proximal and distal coronary openings between felt pledgets (Figs. 4, 5, 6, 7, 8, 9 and 10). Post procedure the patient regained normal sinus rhythm. Pathology of the aneurysm wall revealed calcific atherosclerosis and was negative for vasculitis and connective tissue disease. Her postoperative course was uneventful. She was discharged home on post-operative day five on dual antiplatelet therapy (clopidogrel and aspirin), losartan, and atorvastatin.

## Discussion

CAA is incidentally found in 1.5 to 4.9% of patients undergoing coronary angiography [4]. Giant CAA is even rarer with an incidence of 0.02% [5]. In our patient with a history of an ascending and descending aortic aneurysm, the incidence of a concomitant CAA with presence of one thoracic aneurysm has been reported at 17% [6]. The majority of these CAAs are atherosclerotic in origin [4]. Other causes include congenital heart disease (e.g. Kawasaki‘s disease), trauma, percutaneous coronary intervention, arteritis (e.g. Syphilis, Takayasu), infection (mycotic), and connective tissue disorders. Most CAAs involve the right coronary artery, and the majority are asymptomatic [5, 7].

Diagnosis of CAA is often made by coronary angiography. In our patient, since selective injection of the right coronary artery was unsuccessful, the availability of the multi slice CT angiography (CTA) was utilized. The CTA images obtained visualized the giant RCAA and its compressive effects on the right atrium and ventricle. Patients with CAA may present with angina and myocardial infarction, as did our patient. This is due to myocardial ischemia/infarction from a low flow state, thrombosis, or distal embolization [8]. Other potential complications include rupture, dissection, mechanical compression of adjacent structures, and fistulization into a cardiac chamber [9, 10]. Rupture with pericardial tamponade, if identified, is exceedingly rare and universally fatal [11].

The natural history of CAA and giant CAA are unknown [12]. The pathogenesis of CAA involves the destruction of the vessel media. The resultant thinning of the media, together with increased wall stress, causes progressive dilatation of the segment of the coronary artery as described by Laplace’s law [13]. This results in diffuse coronary ectasia, as well as localized ectatic and aneurysmal segments.

Management of patients with CAA is challenging and is tailored to each patient. Medical therapy for CAA usually consists of antithrombotic and anti-ischemic therapy [14]. There appears to be no significant difference in survival between patients who have aneurysmal or nonaneurysmal coronary artery disease when factors such as hypertension, diabetes mellitus, lipid abnormalities, family history, cigarette smoking, myocardial infarction, and peripheral vascular disease are examined [1]. Percutaneous techniques have been successfully performed; however, long-term outcomes are still unknown [15]. Surgical treatment of giant CAA is advised. Management includes aneurysm ligation, resection, or marsupialization with placement of an interposition graft [16]. However, the most common surgical procedure is opening the CAA, suturing its afferent and efferent vessels, and finishing with bypass grafting of the distal vessel [17]; this procedure was performed in our patient.

## Conclusion

Giant CAA is a rare occurrence. Simultaneous multiple giant coronary aneurysms involving more than one major coronary artery is even rarer. Surgical treatment is recommended in these instances to prevent potential complications.

## Data Availability

Not applicable.

## Abbreviations

CAA: Coronary artery aneurysm
RCA: Right coronary artery
LAD: Left anterior descending coronary artery
CT: Computed tomography
RCAA: Right coronary artery aneurysm
RPDA: Right posterior descending coronary artery
CTA: Computed tomography angiography
